# TEMPO/TCC as a Chemo Selective Alternative for the Oxidation of Hyaluronic Acid

**DOI:** 10.3390/molecules26195963

**Published:** 2021-10-01

**Authors:** Junwen Shan, Thomas Böck, Thorsten Keller, Leonard Forster, Torsten Blunk, Jürgen Groll, Jörg Teßmar

**Affiliations:** 1 Department of Functional Materials in Medicine and Dentistry, Bavarian Polymer Institute (BPI), University of Würzburg, 297070 Würzburg, Germany; junwenshan@gmx.de (J.S.); T_boeck@gmx.de (T.B.); Thorsten.Keller@mail.uni-wuerzburg.de (T.K.); leonard.forster@fmz.uni-wuerzburg.de (L.F.); juergen.groll@fmz.uni-wuerzburg.de (J.G.); 2 Department of Trauma, Hand, Plastic and Reconstructive Surgery, University of Würzburg, Oberdürrbacher Str. 6, 97080 Würzburg, Germany; blunk_t@ukw.de

**Keywords:** hyaluronic acid, oxidation, hydrogel formation, Schiff base chemistry

## Abstract

Hyaluronic acid (HA)-based hydrogels are very commonly applied as cell carriers for different approaches in regenerative medicine. HA itself is a well-studied biomolecule that originates from the physiological extracellular matrix (ECM) of mammalians and, due to its acidic polysaccharide structure, offers many different possibilities for suitable chemical modifications which are necessary to control, for example, network formation. Most of these chemical modifications are performed using the free acid function of the polymer and, additionally, lead to an undesirable breakdown of the biopolymer’s backbone. An alternative modification of the vicinal diol of the glucuronic acid is oxidation with sodium periodate to generate dialdehydes via a ring opening mechanism that can subsequently be further modified or crosslinked via Schiff base chemistry. Since this oxidation causes a structural destruction of the polysaccharide backbone, it was our intention to study a novel synthesis protocol frequently applied to selectively oxidize the C6 hydroxyl group of saccharides. On the basis of this TEMPO/TCC oxidation, we studied an alternative hydrogel platform based on oxidized HA crosslinked using adipic acid dihydrazide as the crosslinker.

## 1. Introduction

Modified biopolymers offer a broad spectrum of hydrogel forming materials that, in recent years, have become increasingly important in the fields of drug delivery, tissue engineering, and regenerative medicine [1,2,3]. As materials from natural origin, they are found in many different mammalian extracellular matrices (ECM), such as HA or collagen, which are derived due to processing slightly degraded gelatin [4,5]. Especially the biotechnologically produced HA and its derivatives are already in clinical use for many specific applications due to their excellent biocompatibility and availability in high purity [6]. For a chemical modification, HA offers several functional groups available for different modification schemes, additionally the polymer is also known to bind to CD44 receptors [7,8,9] and to enhance the chondrogenic differentiation of mesenchymal stem cells in general [10,11].

In the field of tissue engineering, the dissolution stability and mechanical strength of applied biopolymers-based hydrogels is especially crucial for culture and for tissue development under physiological conditions. Without a stable crosslinking, water-soluble polymers will dissolve slowly and accordingly disappear from the application site [12]. Natural HA hydrogels possess only very low mechanical properties as physically crosslinked hydrogels. and therefore are limited in their applications for tissue engineering approaches [13]. To achieve a proper dissolution and mechanical stability, HA needs to be functionalized and, subsequently, chemically crosslinked with other nondetrimental molecules to form long term stable hydrogel networks [14]. For these applications the carboxyl group of HA can be modified, for example, with thiol, tyramine, or dihydrazide, whereas the primary 6-hydroxy groups can be alternatively functionalized with methacrylate and bromoacetate for various chemical or enzymatic crosslinking reactions such as thiol-ene, peroxidase, or photo-crosslinking reactions. These applied modifications of HA have been previously summarized in a review article by Burdick et al. [9]. In addition to the frequently applied chemical networks, there are alternative more stable physical, chemically reversible or irreversible crosslinking mechanisms used for hydrogel formation. For example, physically crosslinked HA hydrogels via a more stable guest–host interaction were introduced by Highley et al., by esterification of the primary alcohol with adamantane acetic acid as guest and amidation of the carboxylate with β-cyclodextrin as host. For the later application in cell culture, they still needed a secondary and irreversible chemical crosslinking by applying radical polymerization of methacrylate, which they attached to non-modified primary alcohols for enhanced mechanical stability [15]. Wang et al., in a similar fashion, introduced HA hydrogels containing adipic acid dihydrazide modified HA as well as periodate oxidized HA (oxHA). This dynamic covalent system, based on reversible Schiff base chemistry, additionally needed chemical fixation by a thiol-ene crosslinked secondary network with norbornene modified HA and a tetrathiol crosslinker to achieve a long-term tissue culture [16].

Many of the physical or dynamically crosslinked hydrogels can also be successfully applied for three-dimensional bioprinting approaches due to their excellent shear thinning properties, but as stated above, those systems require a secondary crosslinking to achieve sufficient mechanical strength and long-term stability for subsequent cell culture. Therefore, other common crosslinking mechanisms, which can also lead to hydrogels without any pre-crosslinking systems, were used to generate irreversible covalent bonds via UV crosslinking, for example, via thiol-ene reaction [17,18] or radical polymerization [19]. However, the UV light used here can harm cells by direct DNA damage, and therefore should be avoided or at least applied with the necessary care [20,21]. In general, therefore, it is desirable to apply mechanisms without any UV crosslinking to provide good cell viability during covalent hydrogel formation. One alternative crosslinking reaction previously described for HA hydrogels is based on Schiff base chemistry, which has been previously reported for pre crosslinking [16,22,23], including different reactive and stable amine derivatives, such as imines, hydrazones, and oximes [24].

For the necessary aldehyde functionalization, one commonly applied method is the oxidation of HA using sodium periodate to generate a dialdehyde in a ring opening reaction of the glucuronic acid unit, which results in a massive alteration of the HA backbone structure. Linkers containing amine, dihydrazide, or aminooxy groups can, subsequently, be used as crosslinkers [22,23,24,25]. Weis et al., showed that hydrogels formed using these materials are not stable for longer than six days in PBS and even less stable in cell culture medium containing free amino acids. At the same time, the degree of oxidation (DO) varies even with same molar stoichiometry and is usually quite moderate, most likely due to numerous side reactions of the oxidation agent periodate [16,22,23]. Accordingly, hydrogels based on Schiff base chemistry using low molecular weight crosslinkers always need a secondary networking, for example, UV crosslinking, to achieve sufficient mechanical and long-term stability [16].

**Scheme 1:**
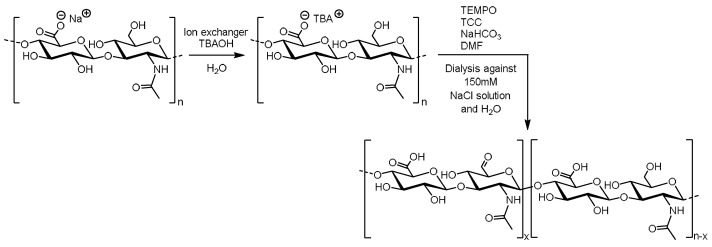
Synthesis of HA with oxidized primary alcohol (proxHA).

A promising alternative to modify HA with aldehyde functionalities is the selective oxidation of the primary alcohol of the *N*-acetylglucosamine unit with (2,2,6,6-tetramethylpiperidin-1-yl)oxyl (TEMPO)/trichloroisocyanuric acid (TCC), as described for the selective oxidation of glycosides. To enable this reaction of HA in DMF, the solubility of HA needs to be enhanced by exchanging the sodium ion of hyaluronan for a tetrabutylammonium ion (Scheme 1). Angelin et al., proved the selectivity of the oxidation to primary alcohols with unprotected monoglycosides with high yields achieved after at least 7 h [26]. For this approach, anhydrous DMF was used as the solvent to avoid overoxidation of the primary alcohol to the carboxylic acid, and sodium bicarbonate was shown to be most efficient to generate the required alkaline conditions. Buffa et al., performed this oxidation in a mixture of water (H_2_O) and dimethylformamide (DMF) to generate degrees of oxidation between 5 and 15%. Additionally, they also tested various secondary oxidation reagents, such as sodium hypochlorite (NaClO) or sodium hypobromite (NaBrO). However, a maximum DO of only 18% was obtained in their studies [27].

In this study, we aimed to oxidize the primary alcohol of HA selectively by using TEMPO/TCC oxidation in anhydrous DMF. We expected preservation of the HA backbone without ring-opening and possibly higher DO. We also assumed that, due to the intact polymer backbone and higher DO, more stable hydrogels could be prepared even in cell culture medium without any secondary crosslinking systems to achieve higher mechanical and potentially long-term stability. Additionally, the reversible chemical crosslinks should allow an erosion of the hydrogels without a chain cleavage catalyzed by hyaluronidase. In these hydrogels, we exemplarily analyzed the chondrogenic differentiation potential of human mesenchymal stromal cells (hMSCs) within these hydrogels. Accordingly, the prepared HA-based hydrogels have already been widely used as favorable matrices in tissue engineering approaches to study the chondrogenic differentiation of encapsulated cells [9,17,28,29]. In our approach, hMSCs were embedded in in situ forming hydrogels and, subsequently, analyzed regarding cell viability and their chondrogenic differentiation potential in vitro. Since it is known that modifications of the HA backbone can affect the binding affinity to CD44 receptors [28], we additionally studied the binding properties of our proxHA and compared this to oxHA and native HA using surface plasmon resonance (SPR) affinity measurements.

## 2. Results

### 2.1. Syntheses and Characterization

**Figure 1:**
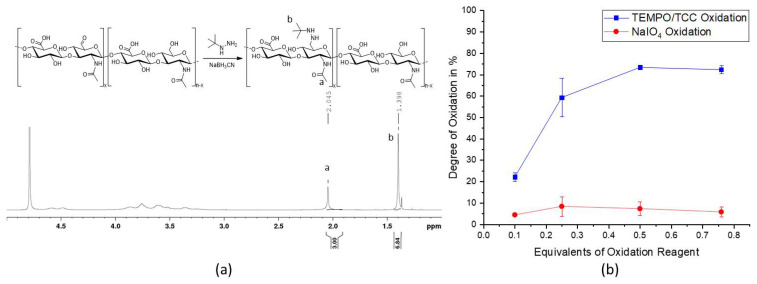
Determination of degree of oxidation (DO) via NMR: (**a**) NMR spectrum of *tert*-butyl hydrazine modified HA measured in D_2_O; (**b**) comparison of TEMPO/TCC and NaIO_4_ oxidized HA in their DO.

The syntheses were processed several times (*n* ≥ 2) for each equivalent of TCC used. To quantify the overall amount of generated aldehyde functions, we stably substituted the aldehyde groups via reductive amination. For this approach, we used amine derivatives which contain a defined number of protons as a *tert*-butyl group and measured NMR spectra of the substituted products (Figure 1a). The acetyl group of the HA dimer unit was calibrated as three protons and the integral of the *tert*-butyl group was divided by the number of *tert*-butyl protons. Consequently, the calculated degree of oxidation refers to the overall amount of oxidized HA dimer units. TCC equivalents of 0.76 and 0.5 led to almost the same DO of 72.7% on average (Figure 1b). The reactions applying 0.25 eq. oxidation reagent led to strong variations of the obtained DOs between 49.5 and 73.7% (Table 1). As compared with the NaIO_4_ oxidation (DO between 4.1 and 11.7%), generally, a much higher DO was achieved with TEMPO catalyzed oxidation with lower amounts of secondary oxidation reagents; even the lowest amount of 0.1 eq. TCC achieved a DO of around 22.2% (Figure 1b). In general, TEMPO/TCC oxidation achieves a three- to twelve-fold higher DO than NaIO_4_ oxidation depending on the equivalents TCC used.

**Figure 2:**
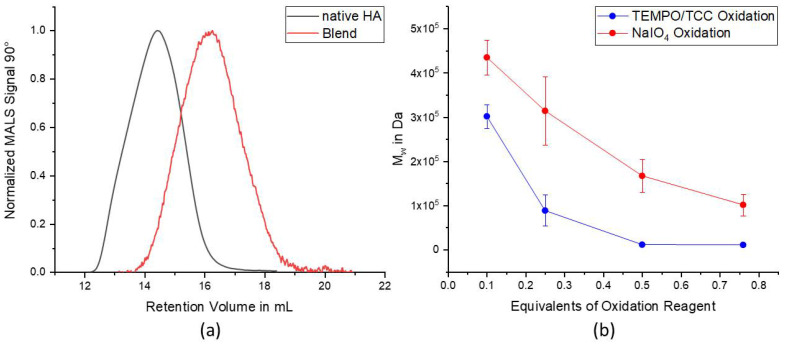
Determination of the molecular weights of the obtained oxidized HA: (**a**) Representative GPC elugrams of the blend as compared with the native HA; (**b**) weight average molecular weight (M_w_) in kDa for the differently oxidized polymers.

Degradation of HA chains during oxidation was investigated by parallel GPC measurements. During TEMPO/TCC oxidation, degradation was also observed as compared with native HA, since the maximum retention volume of the combined oxidized products was higher than the native HA (Figure 2a). As compared with the NaIO_4_ oxidation in which a weight average molecular weight (M_w_) of 101–435 kDa was obtained for the different equivalents of periodate, a pronounced degradation occurs during TEMPO/TCC oxidation with achieved molecular weights of M_w_ 11–302 kDa (Figure 2b). In both cases, we observed increasing molecular weights M_w_ with decreasing amounts of oxidation reagent, which could be attributed to a reduced polymer destruction by the lower amounts of oxidation reagents. Since the DOs for the TEMPO/TCC oxidation were at least three to twelve times higher, we still decided to investigate the influence of the obtained individual oxidation degrees on hydrogel stiffness and incubation stability.

### 2.2. Hydrogel Formation

To investigate the influence of oxidation degree on hydrogel formation, we used different batches with the DOs of 73.7. 57.2, 55.0, and 49.5%. Additionally, we combined batches with similar DOs of 57.2, 55.0, and 49.5% to a mixed blend and compared the hydrogels made out of the blend and the single batches at a final content of 3% (*w*/*w*) for their hydrogel formation. We also analyzed the effect of increasing polymer content on the mechanical properties of the hydrogels generated with the blend by using final contents of 2, 3, 4, and 5% (*w*/*w*).

#### 2.2.1. Compression Tests for Young’s Modulus

**Figure 3:**
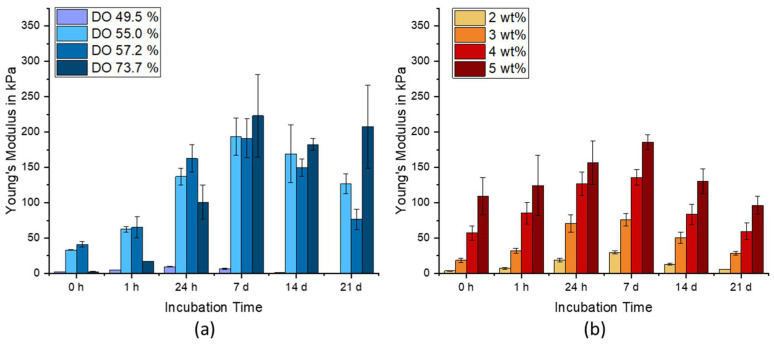
Young’s moduli of: (**a**) Various batches with DO 49.5%, 55.0%, 57.2%, and 73.7% and a polymer content of 3 wt%; (**b**) the mixed polymer blend (DO 53.3%).

Right after preparation, the batch with the lowest DO also showed the weakest mechanical properties, as indicated by the measured stiffness (2.4 ± 0.04 kPa), whereas the batches with DO 55.0% (33.2 ± 0.7 kPa) and 57.2% (40.9 ± 3.9 kPa) showed significantly higher Young’s modulus values (Figure 3a). After 14 days of incubation, the DO 49.5% hydrogels were already completely swollen and dissolved. The batch with the highest investigated DO 73.7% first showed Young’s modulus values similar to the hydrogels with DO 49.5% (2.5 ± 0.7 kPa on day 1), but the stiffness of these hydrogels increased very rapidly as compared with the other batches due to significant contraction, and finally achieved the highest mechanical strength after 7 d with 222.9 ± 58.3 kPa. The hydrogels from the other batches with DO 55.0% (193.3 ± 26.3 kPa) and DO 57.2% (191.03 ± 27.46 kPa), after seven days, also achieved their maximum Young’s modulus values. This effect of increasing Young’s modulus values during long-term storage in PBS could be observed for all single batches as well as the mixed blend. After Day 7, almost all hydrogel formulations lost the mechanical strength and became weaker again.

Hydrogels made from the blend in different contents proved that Young’s modulus values could be further increased with higher polymer content (Figure 3b). Overall, on day seven, the 2% (*w*/*w*) hydrogels showed Young’s modulus values six times lower than those of the 5% (*w*/*w*) hydrogels and even 31 times lower than on day one. The 3% (*w*/*w*) hydrogels of the blend appeared at least almost two times less stiff as compared with the hydrogels of the single batches DO 55.0% and DO 57.2% on day one, which could be attributed to the content of lower oxidized HA. At the maximum of mechanical strength on day seven, the 3% (*w*/*w*) blend hydrogels with 76.1 ± 8.6 kPa showed Young’s modulus values 2.5 times lower than the hydrogels made from the batches with DOs 55.0% and 57.2%. All hydrogels achieved their maximum stiffness at day seven and lost mechanical strength afterwards.

#### 2.2.2. Swelling and Degradation Behavior

**Figure 4:**
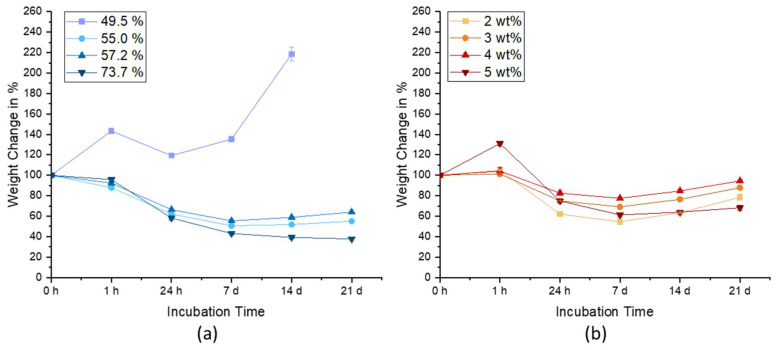
Behavior of hydrogels after storage in 1 mL PBS: (**a**) Various batches with DO 49.5%, 55.0%, 57.2%, and 73.7% with a content of 3 wt%; (**b**) blend (DO 53.3%) with various contents.

In addition to the mechanical properties, weight changes of the hydrogels during storage in PBS were also investigated. Here, we observed a shrinkage of all hydrogels based on polymers with a higher DO then 50% (Figure 4a) and content of 3 wt% polymer. After 21 d, the hydrogels shrank to about 55.1 ± 0.2% of their initial weight for DO 55.0%, to 64.0 ± 1.1% for DO 57.3% and even to less than the half of the initial weight (39.3 ± 1.4%) for DO 73.7%. For the lower oxidation (49.5%), the hydrogels initially swelled to 143.4 ± 2.6% of the starting weight, shrank after 1 d to 119.2 ± 0.9%, and swelled afterwards until complete dissolution. The blend hydrogels did not shrink after 1 h but swelled all over 100% (Figure 4b), then, the resulting shrinkage was quite moderate as compared with the single batches and stopped between 68.3 ± 2.3% (5 wt%) and 94.6 ± 1.3% (4 wt%).

### 2.3. SPR Analysis for Determination of Binding Affinity of ProxHA

**Figure 5:**
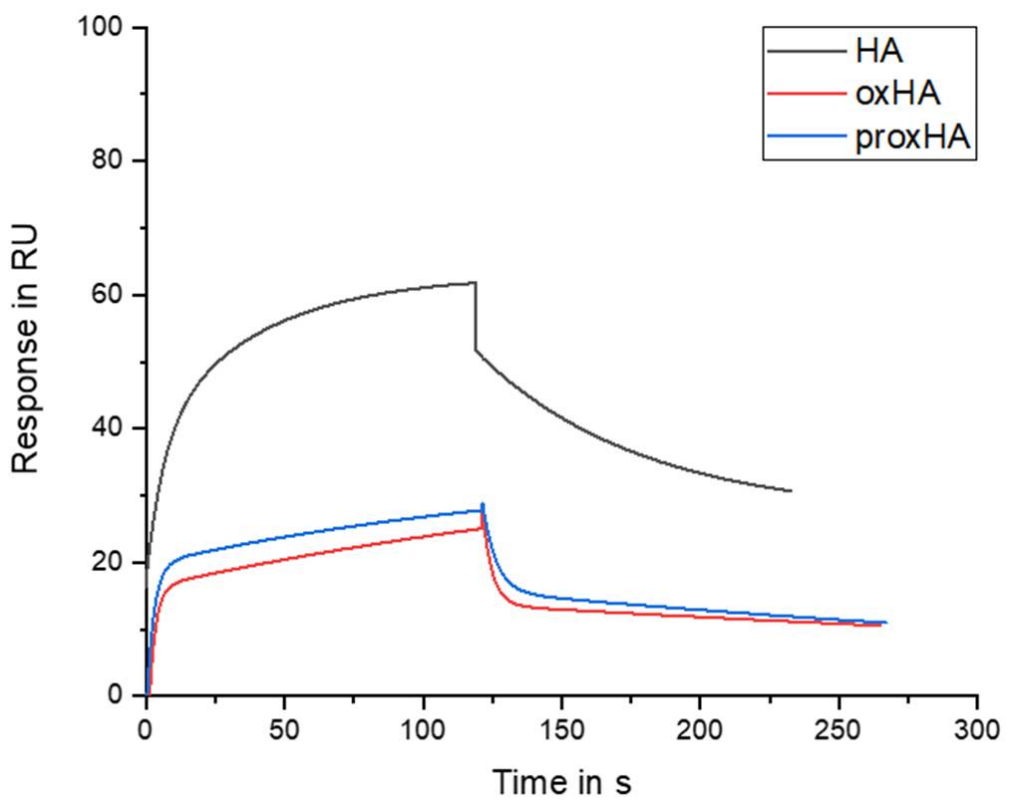
SPR analysis of: (black) unmodified HA; (red) NaIO_4_ oxidized (oxHA); (blue) TEMPO/TCC oxidized (proxHA), at the highest investigated concentration of 32.7 µM.

Since chemical modifications of HA can strongly influence its binding properties to the CD44 receptor on cell surfaces [28], we studied their binding to surface immobilized CD44 receptors via SPR measurements. In this study, we investigated and compared the CD44 binding of unmodified HA (HA, M_n_ = 24 kDa), the NaIO_4_ oxidized HA (oxHA, M_n_ = 121 kDa, DO 11.7%), as well as the primary oxidized HA (proxHA, M_n_ = 11 kDa, DO 63.2%) oxidized with the same equivalents of the respective oxidation agents. Despite the fact that proxHA has an almost six-fold higher DO than oxHA, and a ten-fold lower molecular weight, both *K_D_* values were still in a similar range, which can be attributed to the intact backbone structure of the proxHA favoring the interaction with the CD44 receptors and the retention of the polymer on the SPR chip (Table 2). However, both the oxidation of the primary alcohol to an aldehyde function and the formation of the dialdehyde seemed to have a major effect on the binding ability as compared with the native HA of a comparable molecular weight (Figure 5).

### 2.4. Cell Viability of hMSCs

To evaluate the in vitro cytocompatibility of the obtained hydrogels, hMSCs were embedded in hydrogels formed out of the polymer blend to investigate the effect of polymer contents (3, 4, or 5 wt%) on cell survival and differentiation in proliferation medium as well as in chondrogenic differentiation medium for 21 days. Depending on the chosen type of culture medium, there was quite a remarkable difference in hydrogel stability. In proliferation medium, the hydrogels with 3 and 4 wt% polymer both dissolved between day 7 and day 21, whereas the 5 wt% blend hydrogels did not disintegrate but became slightly larger during in vitro culture. Additional cell-containing hydrogels (3 wt%) based on polymers with a DO of 73.7% retained their size in cell culture and remained stable even after Day 21 (Figure S1). In contrast, all the hydrogels that were cultured in chondrogenic differentiation medium did not dissolve at all but decreased in size, with 5 wt% hydrogels showing the strongest shrinkage of all studied formulations (Figure S2).

**Figure 6:**
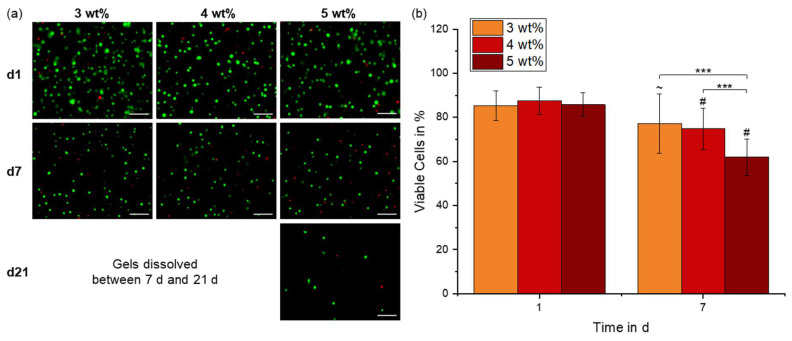
Cell viability in blend hydrogels: (**a**) Cell viability of in vitro cultured hMSCs in 3, 4, and 5 wt% blend hydrogels (DO 53.3%) after days 1, 7, and 21 in proliferation medium, shown by live/dead staining. Viable cells were labeled green with calcein-AM and dead cells with EthD-I. Scale bars represent 100 µm; (**b**) Quantification of viable cells, after days 1 and 7 of in vitro culture in proliferation medium. Data are presented as means ± standard deviation (*n* = 3 per condition and time point, counting at least eight locations for each sample). Significant differences between groups at day 7 are marked with *** (*p* < 0.001), labels above bars of groups at day 7 show significant differences as compared with day 1. “~” *p* < 0.01 and “^#^” *p* < 0.001.

Live/dead assays were performed for all polymer contents of blend hydrogels and showed excellent biocompatibility (viable cells > 85%) of all investigated hydrogels at day 1 (Figure 6a,b). On day 7, only a slight decrease in cell viability was observed for 3 wt% and 4 wt% hydrogels (77.2 ± 13.5 and 74.8 ± 9.5%, respectively), whereas significantly less viable cells (61.9 ± 8.3%) were detected for 5 wt% gels (Figure 6b).

### 2.5. Chondrogenic Differentiation of hMSCs

**Figure 7:**
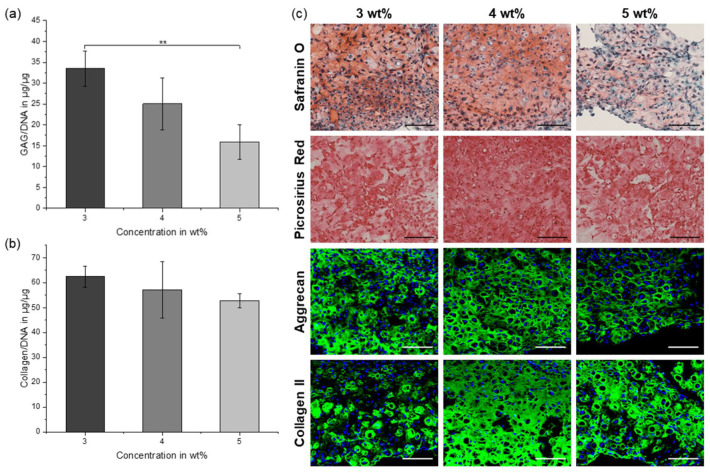
(**a**) GAG; (**b**) total collagen production of in vitro cultured hMSCs in 3, 4, and 5 wt% blend hydrogels (DO 53.3%) after 21 days of chondrogenic differentiation, normalized to DNA. Data are presented as means ± standard deviation (n ≥ 3). Significant differences between groups are marked with ** (*p* < 0.01); (**c**) histological and immunohistochemical staining for deposited ECM components in 3, 4, and 5 wt% blend hydrogels (DO 53.3%) after 21 days of chondrogenic differentiation. Longitudinal sections were stained for deposition of GAG and collagen with safranin O and picrosirius red, respectively. Immunohistochemical staining for aggrecan (green) and collagen type II (green), nuclei were counterstained with DAPI (blue). Scale bars represent 100 µm.

After culture of hMSCs in the chondrogenic differentiation medium, the deposition of cartilaginous ECM within the hydrogels was assessed. Quantitative biochemical analysis of glycosaminoglycan (GAG) production showed that hMSCs encapsulated in the lowest concentrated hydrogels (3 wt%) produced distinctly higher amounts of GAG/DNA as compared with 5 wt% hydrogels (15.9 ± 4.1 µg/µg) (Figure 7a). This could also be demonstrated by histological staining with safranin O for GAG and immunohistochemical staining for aggrecan, where the 5 wt% hydrogels exhibited the weakest signals for these cartilage-specific ECM molecules (Figure 7c). The staining for collagens with picrosirius red and collagen II, on the other hand, did not show differences in collagen production between the different hydrogel formulations (Figure 7c). Furthermore, these findings were reflected by the determination of the total collagen/DNA ratio, with only slightly, but not significantly lower values for 5 wt% as compared with 3 wt% (Figure 7b). Altogether, the assays confirmed that considerable ECM production during chondrogenic differentiation was achieved in all tested hydrogels and that the lowest concentrated 3 wt% hydrogels showed higher GAG production as compared with the highest concentrated hydrogels.

## 3. Discussion

According to this study, we established a new synthesis path of a primary oxidized HA for the formation of dynamic chemically crosslinked hydrogels with high mechanical strength and long-term stability for cell culture. Additionally, we optimized the determination method for the aldehyde content of oxidized HA and other comparable biopolymers. In studies in the literature, *tert*-butyl carbazate, a BOC-protected hydrazine, is often used for the quantitative analysis [23], but we found out that the BOC protection group can already be cleaved under the mild acidic conditions during the modification [30]. Since the reductive amination is performed in acetate buffer with pH 5.2, the use of *tert*-butyl carbazate can deceive the obtained DO. With *tert*-butyl hydrazine, the cleavage of the *tert*-butyl group used for quantification is avoided and reliable results are obtained.

**Scheme 2:**
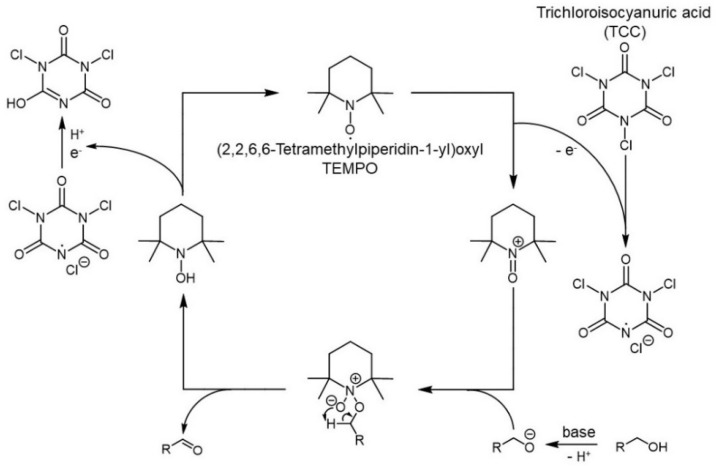
Mechanism of oxidation of primary alcohol with TEMPO/TCC.

During the characterization of proxHA using TBH, we observed that both higher TCC equivalents of 0.76 and 0.5 led to a similar DO over 70%. The oxidation of the primary alcohol of HA is performed by TEMPO which is acting as catalyst and needs activation and regeneration by a secondary oxidation reagent. For this, TCC is used both to activate and regenerate TEMPO in a two-step oxidation [26]. Since all three chlorine atoms are active, TCC can function three times as oxidation reagent and finally convert to cyanuric acid with an aromatic ring, which is the driving force for the reaction [31] (Scheme 2). Additionally, the reaction must be performed under strictly anhydrous conditions to avoid subsequent oxidation to carboxylic acid functions [26]. For this reason, 0.5 and 0.76 equivalents TCC should be able to convert nearly all primary alcohol groups of N-acetyl-D-glucosamine during the reaction. Still, no complete conversion of all HA dimer units was achieved, therefore, we assume that the oxidation limit could capped at a certain degree of oxidation due to steric effects, since the obtained degrees of oxidation for higher amounts of TCC were quite similar (Figure 1b). Taking the three active chlorine species into account, it was expected to achieve a DO of over 70% already with 0.25 eq. of TCC, which occurred only once with DO 73.7% due to excellent reaction conditions. Since HA is a very sensitive and eventually also hygroscopic biomacromolecule, reproducibility of the reactions with HA is often difficult to accomplish. Under best conditions, a DO of around 60% was achieved on average and this is, nonetheless, 2.4 times higher than the equivalents of added TCC. A similar effect was observed for the reactions with only 0.1 equivalents of TCC, indicating the multiple step oxidation of the secondary oxidation reagent. As compared with the oxidation with NaIO_4_, Dos that were at least 3–12 times higher were obtained depending on the used equivalents of TCC. Unfortunately, the GPC measurements, nevertheless, also showed a massive degradation of the HA chains after TEMPO/TCC oxidation. However, it is well known that degradation of HA chains can occur under oxidative stress (e.g., caused by free radicals) or under non physiological pH conditions both during chemical reactions as well as in biological systems [32,33,34,35].

When using the polymers for the formulation of hydrogel, we observed very fast gelation with adipic acid dihydrazide, within 10 s or faster, especially with higher polymer contents and oxidation degrees. Due to the immediate gelation, the obtained hydrogels were quite stiff and brittle, therefore, we assume that these hydrogel systems cannot be used for extrusion-based 3D bioprinting. Since a higher DO led to more crosslinking and, consequently, higher network density, it is also expected that a higher DO would result in a higher Young’s modulus value. Upon incubation in PBS, we observed increased Young’s modulus values of almost all hydrogels, except the hydrogels with DO 49.5%, which even dissolved completely between Days 14 and 21, due to the lowest DO and resulting low crosslinking efficiency. One possible explanation for this increase in the value of Young’s modulus is the dynamic reversible bond of the Schiff base chemistry, which can be rearranged due to the reversibility until the most stable thermodynamic state and, consequently, highest mechanical stability is reached. Additionally, we assume that the shrinkage of the hydrogels that occurs after storage in PBS is also related to this rearrangement of the network. The higher hydrogel network density caused by syneresis again explains the occurring increase in the mechanical strength at the later timepoints. A similar effect of higher network density can also be achieved by increasing the content of the hydrogel forming polymer solutions, which is demonstrated with the blend hydrogels formed by different polymer contents (Figure 3b). As compared to the individual polymer batches, the blend hydrogels showed a reduced mechanical strength and rather moderate swelling behavior, which can be attributed to the overall mixed behavior of the differently oxidized polymer batches. Accordingly, the behavior of the hydrogels can be tuned by the oxidation degree of the used polymers. In general, the formed hydrogels with an average oxidation degree above 50%, proved to be stable for extended periods of time even without cells (over 3 weeks), which was never been achieved with hydrogels based on NaIO_4_ oxidized HA and the low molecular weight crosslinker ADH [22]. As compared with other HA hydrogels crosslinked only via reversible Schiff base, for example, the hydrazine modified HA crosslinked with NaIO_4_ oxidized HA [16], our proxHA hydrogels are also mechanically much stiffer. We presume this is mainly induced by the high degree of oxidation and eventually also the preservation of the HA backbone structure.

Since we only modified the primary alcohol of HA without changing the backbone structure excessively, we were interested in the receptor binding affinity of our HA derivative as compared with the commonly performed HA oxidation using periodate. Bhattacharya et al., summarized the interactions between the functional groups of HA and the CD44 receptor, and showed that hydrogen bonds between the charged glucuronic acid and the amino acids predominate the hydrogen bonds and hydrophobic interaction of the N-acetylglucosamine [28,36]. Oxidation with NaIO_4_ obviously leads to an opening of saccharide rings, which causes an extreme interference with the retention of HA backbone structure. We assumed that this interference by ring opening, and oxidation has a much higher influence on the CD44 receptor binding properties of HA than the oxidation of the primary alcohol group to an aldehyde function. However, both binding affinities of the differently oxidized HA derivatives were significantly lower than the native HA, which demonstrates the specificity of CD44 for the preserved structure of HA. Despite the fact that NaIO_4_ oxidized HA only had an almost six-fold lower DO and a ten-fold higher molecular weight than proxHA, the *K_D_* values of both oxidized HA were in a similar range. This demonstrates that even with preservation of the backbone the high DO negatively affects the binding affinity of the HA derivative (Table 2). However, given the fact that the CD44 receptor needs at least four intact HA repeating units for binding [8], which is achievable by oxHA with a DO of 11.7% (every 10th repeating unit is oxidized), it is impossible to find as much unchanged repeating units in the proxHA, where six out of ten repeating units are already oxidized. Accordingly, we suppose that the NaIO_4_ oxidation massively destroys the backbone structure, and thereby interferes with the receptor interaction leading to the weak binding affinity even with a lower oxidation degree. The exchange of the primary alcohol to an aldehyde function, accordingly, seems to have a minor effect on the binding ability (Figure 5) in this study.

Further striking differences were observed during the in vitro cell culture in the formed hydrogels. The hydrogels were only allowed to crosslink for 30 min, since the supply of cells with nutrients should not be disrupted for too long. Without storage in any aqueous solutions, there is no possibility for the hydrogels to absorb additional water for enhancement of the dynamic Schiff base formation. Here, it was observed that the hydrogels formed out of 3 and 4 wt% polymer dissolved completely during in vitro culture in proliferation medium, which was most likely caused by the reversible dynamic Schiff base system where the formed hydrazones undergo hydrolysis [37]. Additionally, the dissolution of the hydrogels was possibly enhanced by the presence of amino acids in cell culture medium, which can obviously replace ADH as a bifunctional crosslinker. In strong contrast, all hydrogels which underwent differentiation retained their stability in cell culture, which we attributed to the newly formed ECM by the hMSCs, which was most likely integrated in the reversible hydrogel network. Nevertheless, a higher DO could still be employed to improve stability due to more crosslinking possibilities for ADH, and thereby also allow a longer culture for undifferentiated cells (Figure S2). In general, all hMSCs (undifferentiated and differentiated) within the hydrogels initially showed an excellent cell viability with over 85% after encapsulation. The viability of cells decreased after seven days, especially for the higher polymer containing 5 wt% hydrogels (Figure 6b), which was also observed in other HA-based hydrogels and could probably be caused by limitations of nutrient and waste diffusion due to the denser polymer network [38,39,40]. The toxicity of the also present low molecular weight crosslinker was only minute as demonstrated in cell culture studies (data not shown).

Furthermore, the deposition of GAG and collagen throughout the hydrogel matrix proved the formation of cartilage specific ECM [41]. The significant reduction in the GAG/DNA ratio in 5 wt% hydrogels as compared with lower concentrated hydrogels, i.e., 3 wt% (Figure 7a), was in line with reports for other HA hydrogels which showed that higher polymer content can reduce GAG production of encapsulated cells [42,43,44]. In addition to this, the total collagen content did not indicate significant differences between the different contents (Figure 7b). The considerable shrinkage of hydrogels cultured under chondrogenic conditions after 21 days, especially for high concentrated gels with less ECM production (Figure S2), could also be explained with the dynamic bonds of the Schiff base chemistry, which allow a steady replacement of the ADH crosslinker by the ECM produced by the cells, and thereby a further contraction or condensation of the hydrogel. Here, the reversible Schiff base chemistry also enabled a continuous remodeling of the hydrogels without any necessary cleavable crosslinkers. Additional histological and IHC staining, accordingly, showed an aggregation of cells and homogeneous distribution of cartilaginous matrix components such as GAG, aggrecan, and collagen II, especially throughout the lower concentrated hydrogels, providing more space for the formed ECM components (Figure 7c). The deposition of cellular ECM in these reversibly crosslinked hydrogels appeared to be more even as compared with other HA hydrogel systems [17,42,43]. Taken together, in this study, the presented hydrogel system based on highly oxidized HA seems to be well suited for the culture of ECM forming cells, especially hMSCs undergoing chondrogenesis in this pilot experiment.

## 4. Materials and Methods

### 4.1. Materials

HA (1.0–2.0 MDa, 80–100 kDa), 1-ethyl-3-(3-dimethylaminopropyl) carbodiimide (EDC), N-hydroxysuccinimide (NHS) were purchased from Carbosynth Limited (Berkshire, UK). Adipic acid dihydrazide (ADH), anhydrous dimethylformamide (DMF), dimethylhydrazine, DOWEX^®^ 50WX8-400 ion exchanger, Dulbecco’s phosphate buffered saline (DPBS), ethidium homodimer-I (EthD-I), sodium azide, sodium chloride, sodium cyanoborohydride, sodium periodate, *tert*-butyl hydrazine hydrochloride (TBH), tetrabutylammonium (TBA) hydroxide solution (~40% in water), and trichloroisocyanuric acid (TCC) were purchased from Sigma-Aldrich, St. Louis, MO, USA. 2,2,6,6-Tetramethylpiperidine-1-oxyl (TEMPO) was acquired from Alfa Aesar (Karlsruhe, Germany). Human CD44 Fc Chimera Recombinant Protein was obtained from R&D Systems (Minneapolis, MN, USA). The acetic acid, ethylene glycol, potassium chloride, and sodium nitrate were purchased from Merck KGaA (Darmstadt, Germany). Proteinase K was purchased from Merck Millipore (Burlington, MA, USA). Calcein-acetoxymethyl ester (calcein-AM), Dulbecco’s modified Eagle’s medium (DMEM), 0.25% trypsin-EDTA, HEPES 1M, penicillin-streptomycin, Quant-iT™ PicoGreen^®^ dsDNA Reagent and Kit, and sodium hydrogencarbonate were acquired from Thermo Fisher Scientific (Waltham, MA, USA). ROTI^®^Histofix 4.5% and ZelluTrans/Roth dialysis tubing (regenerated cellulose, MWCO 3500 Da) were purchased from Carl Roth GmbH and Co. KG, Karlsruhe, Germany.

### 4.2. Syntheses of Tetrabutylammonium Hyaluronate (TBA-HA)

The transformation of sodium hyaluronate into tetrabutylammonium hyaluronate (TBA-HA) was performed, according to Oudshoorn et al., with slight modifications [45]. The exchange of the sodium counterions was performed with 50 g DOWEX^®^ 50WX8-400. The resin was washed three times with 1 L Millipore H_2_O and, afterwards, suspended in 98 mL TBA hydroxide solution, stirred for two hours at room temperature (RT), and then separated from the solution by filtration. For the preparation of TBA-HA, 5 g HA was dissolved in 600 mL Millipore water overnight at RT, and then suspended with 50 g resin. The suspension was shaken for 3 h and the resin was removed by centrifugation. TBA-HA was obtained by lyophilization (Epsilon 2–4 LSCplus, Martin Christ Gefriertrocknungsanlage GmbH, Osterode, Germany).

### 4.3. Syntheses of Primary Oxidized Hyaluronic Acid (proxHA)

All subsequently used equivalents were calculated corresponding to one repeating unit of the TBA-HA (M = 621.79 g/mol). TBA-HA was dissolved overnight at RT in anhydrous DMF (0.5% *w*/*v*) under inert gas. TEMPO (0.0513 eq., catalytical amount) and excess of solid NaHCO_3_ (30 eq.) were added. Then, the suspension was cooled to 4 °C and different amounts of TCC (0.76, 0.5, 0.25, and 0.1 eq.) were added. The reaction was subsequently stirred for 4 h, and then slowly warmed to RT. Afterwards, an excess of ethylene glycol (30 mL for 750 mg TBA-HA and 0.25 eq. of TCC) was added to quench the reaction and remaining oxidative potential. Then, the obtained reaction mixture was dialyzed against 150 mM NaCl for 2 d, and then against Millipore H_2_O for another 2 d (MWCO 3500 Da). The final modified polymer was obtained after lyophilization as a white solid foam. For cell studies and mechanical testing, several batches obtained with 0.25 eq. of TCC were consolidated to receive one larger blend batch to achieve sufficient reproducibility of subsequent experiments and to investigate the influence of batch-to-batch variations.

### 4.4. Syntheses of Hyaluronic Acid Dialdehyde (oxHA)

The synthesis was performed according to Jia et al., with slight modifications [23]. HA was dissolved in Millipore H_2_O overnight. A 0.4 M sodium periodate solution was added dropwise under exclusion of light with the following equivalents: 0.76, 0.5, 0.25, and 0.1. The reaction was stirred for 2 h at RT and similarly quenched by adding 30 mL ethylene glycol. The oxHA was obtained after dialysis (MWCO 3500 Da) against ddH_2_O and lyophilization as a white solid foam.

### 4.5. Determination of the Degree of Oxidation (DO)

Aldehyde containing HA was dissolved in acetate buffer (pH 5.2) overnight at RT. Stock solutions of *tert*-butyl hydrazine hydrochloride and sodium cyanoborohydride (0.5 m) were added. The mixtures were shaken at RT for 24 h at 850 rpm. Then, the solutions were dialyzed against Millipore H_2_O (MWCO 3500) and lyophilized. ^1^H-NMR spectra of the products were measured with 300 MHz NMR (Bruker NMR Fourier 300) in D_2_O. The acetyl group of HA (2.05 ppm) was calibrated as 3 protons. The *tert*-butyl signal (1.40 ppm) of each sample was accordingly divided by 9 protons to estimate the achieved degree of oxidation (DO) as follows:

(1) DO % =NMR integral of Btu9·100

### 4.6. Gel Permeation Chromatography (GPC)

Weight and number average molecular weights (M_w_ and M_n_) of proxHA and oxHA were determined by using a GPC system from Malvern (Herrenberg, Germany). The system consisted of a Viscotek GPCmax (in-line degasser, 2-piston pump and autosampler), a column oven (35 °C) refractive index (RI) detector (Viscotek VE3580), multiple angle light scattering detector (Viscotek SEC-MALS 20, laser wavelength 660 nm), and two Viscotek A-columns (A6000M, length = 300 mm, width = 8 mm, porous poly(methyl methacrylate), particle size 13 µm). An aqueous solution of Millipore H_2_O with 8.5 g/L NaNO_3_ and 0.2 g/L NaN_3_ was used as eluent and solvent for the polymers. The samples were dissolved with a concentration of 0.5 mg/mL overnight at RT and filtered with a 0.45 µm regenerated cellulose membrane. The measurements were performed with 100 µL injection volume with an elution rate of 0.7 mL/min. The molecular weights of the HA derivatives were calculated using a MALS calibration performed with a narrowly distributed PEG standard of M_w_ = 45,000.

### 4.7. SPR Measurements for Binding Affinity of HA Derivatives

Interactions between the differently modified HA and the corresponding HA receptor CD44 (R&D Systems, Inc., Minneapolis, MN, USA) were measured by SPR using a Reichert^®^ 4SPR system (Reichert Technologies, Unterschleissheim, Germany). For immobilization of the CD44 receptor on the sensor surface, the carboxylate groups of a carboxymethyldextran hydrogel coated sensor chip (XanTec bioanalytics GmbH, Düsseldorf, Germany) were activated, first, by perfusing a 1:1 mixture of NHS and EDC (1-ethyl-3-(3-dimethylaminopropyl) carbodiimide), according to the manufacturer’s recommendation. Then, CD44 (10 µg/mL in 10 mM NaOAc, pH 4.5) was injected onto the activated sensor for 8 min at a flow rate of 10 µL/min to cause CD44 coating to a final density of 2000 resonance units. Unreacted activated carboxylate groups were quenched by injecting 1 M ethanolamine (pH 8.5) for 300 s. Afterwards, measurements were performed at 25 °C using HBS150T (10 mM HEPES (pH 7.4), 150 mM NaCl, 3.4 mM EDTA, 0.005% (*v*/*v*) Tween 20) as running buffer. Native HA or oxidized HA derivatives were used as analyte at four different concentrations, ranging from 32.7 to 2.7 µM. The flowrate for the acquisition of interaction data was set to 25 µL/min in all experiments. Association was measured for 120 s, then, dissociation was initiated by perfusing running buffer, and the dissociation phase was monitored for 300 s. Regeneration of the chip surface was performed by injecting of two 60 s pulses of 100 mM glycine (pH 2.5) at a flow rate of 100 µL/min. Interaction data were analyzed using the software TraceDrawer version 1.8.1 (Ridgeview Instruments AB, Uppsala, Sweden), applying a simple Langmuir-type 1:1 interaction model and using global fitting for the rate constants. Association rate constant *k_on_* values and dissociation rates constant *k_off_* values were obtained by fitting data of individual experiments. Equilibrium binding *K_D_* values were deduced using the equation:

(2) $$K_{D}=\frac{k_{off}}{k_{on}}$$

All SPR experiments were performed in at least three independent experiments.

### 4.8. Hydrogel Preparation

Hydrogels were generally prepared in PBS with final polymer contents of 2, 3, 4 and 5% (*w*/*w*) for proxHA (Table S1). The polymers were dissolved for 3 h at 37 °C and the 1 or 2% (*w*/*w*) ADH in PBS was also prepared fresh for every experiment. For all hydrogels, equimolar ratios of aldehyde to hydrazide were used. The ADH solution was added to the polymer solution, thoroughly mixed with the pipette and then the solution was immediately transferred to appropriate cylindrical molds. The gels were allowed to cross-link in petri dishes with added wet tissues to avoid drying out, the 3, 4 and 5 wt% hydrogels for 1 h and the 2% hydrogels for 2 h to achieve sufficient crosslinking before adding them into the incubation media.

### 4.9. Mechanical Testing

For mechanical testing, as well as the swelling/degradation behavior, hydrogels were prepared in PBS as described in Section 4.8 using cylindrical molds (4 mm diameter, 4 mm height). Mechanical strength was measured from the resulting hydrogels directly after preparation and after storage in 1 mL PBS for 1 h, and 24 h, 7 days, 14 days, and 21 days. The linear compression tests were performed with a mechanical test instrument (ElectroForce 5500, TA Instruments, Eden Prairie, MN, USA) with a load cell of 250 g and a rate of 0.005 mm/s. The height and diameter of each sample were measured with a sliding caliper before each mechanical testing. The strain *γ* was calculated by dividing displacement (disp) of the measurement by the height *h*_0_, as shown Equation (3):

(3) $$\gamma =\frac{displ}{h_{0}}$$

True stress was calculated as follows:

(4) $$True\;stress=load\;\left(N\right)\;\frac{h_{0}-disp}{V_{sample}}$$

By plotting strain versus true stress and using the linear fit of the strain ranges showing, Young’s modulus was obtained from the slope of the linear fit. On the basis of the incubation period of the hydrogels, the compression depth and the strain ranges for the calculation of Young’s modulus were adjusted, due to the contraction of the hydrogels (0 and 1 h: l = 1 mm, strain 10 to 20%; all other time points: l = 0.5 mm, strain 2.5 to 12.5%).

### 4.10. Swelling and Degradation Behavior

To observe the swelling or degradation behavior of the hydrogels after incubation in PBS, the hydrogels were taken out of the solution after 1 h, 24 h, 7 d, 14 d, and 21 d; blotted dry using analytical tissues; weighed; and compared to initial hydrogels at time 0 h. The ratios of the weight of incubated hydrogels (*w_s_*) and weight of non-incubated hydrogels (*w*) were calculated using the following equation:

(5) $$WB=\frac{w_{s}}{w}$$

### 4.11. Cell Culture

Human bone marrow-derived mesenchymal stromal cells (hMSC) were used for biological evaluation of the obtained hydrogels. The hMSC were isolated from the cancellous bone of patients undergoing hip replacement (as approved by the Local Ethics Committee of the University of Wuerzburg (186/18) with the written informed consent of each donor patient.) The hMSC were extracted by extensively washing of the bone fragments and bone marrow with PBS. Following this, the cell containing suspension was centrifuged and the obtained cell pellet was resuspended in proliferation medium (DMEM/F12, supplemented with 10% FCS, 1% PS, 50 μg/mL L-ascorbic acid, 2-phosphate sesquimagnesium salt hydrate, and 5 ng/mL bFGF (BioLegend, London, UK)) and seeded into T175 cm^2^ flasks (Greiner Bio-One, Frickenhausen, Germany). After several days the nonadherent cells were removed by washing carefully with PBS and adherent cells were cultured to a sub-confluent level at 37 °C and 5% CO_2_ in proliferation medium. Finally, MSCs were detached with 0.25% trypsin-EDTA and seeded at a density of 3–5×10^4^ cells mL^−1^ into T175 cm^2^ flasks.

### 4.12. MSC Encapsulation in Blend Hydrogels

Blend hydrogels were prepared as described in Section 4.8 for encapsulation of MSCs. Briefly, proxHA was sterilized using germicidal UV light at 254 nm for 20 min (UVL hand lamp with filter, A. Hartenstein, Wuerzburg, Germany). Afterwards, the proxHA was dissolved in PBS to achieve final polymer contents of 3, 4, and 5% (Table S2). The ADH solution was sterile filtered through a 0.2 µm syringe filter.

Initially, MSCs at Passage 4 were resuspended in the corresponding blend proxHA solutions. Then, the cell-laden proxHA solution was mixed with the ADH solution separately for each individual hydrogel and immediately transferred into a silicon mold with 6 mm diameter and 2 mm height (60 µL). The hydrogel precursor solutions were allowed to gel for 30 min at 37 °C and 5% CO_2_ in humidified environment. Then, the hydrogels were transferred into the cell culture medium using a spatula and were cultivated in vitro for 21 days either in proliferation medium or in chondrogenic medium (Dulbecco’s modified Eagle’s medium high glucose 4.5 g/L (DMEM) supplemented with 1% ITS + Premix (Corning, NY, USA), 40 μg/mL L-proline, 50 μg/mL L-ascorbic acid 2-phosphate sesquimagnesium salt hydrate, 0.1 μM dexamethasone, 1 mM sodium pyruvate, 1% PS, and 10 ng/mL transforming growth factor-β1 (TGF-β1, BioLegend, London, UK)).

### 4.13. Cell Viability Assay

The viability of encapsulated MSCs cultivated in proliferation medium was analyzed on days 1, 7, and 21 using calcein acetoxymethyl ester (calcein-AM) to detect viable cells and ethidium homodimer-I (EthD-I) for the detection of dead cells. Therefore, the cell containing hydrogels were washed two times with PBS and were subsequently incubated for 45 min at RT in the staining solution (1 µM EthD-I, 2 µM calcein-AM). Then, the constructs were washed with PBS and top view images were taken using a fluorescence microscope (Axio Observer Z1, equipped with epifluorescence optics and an MRm camera, Carl Zeiss, Jena, Germany). The ratio of viable and dead cells was determined for three samples per condition, counting at least at eight different locations within each sample using “Find Maxima” (settings for “live” and “dead” channel: prominence = 75, strict, exclude edge maxima, output = count) with Fiji [46].

### 4.14. Histology and Immunohistochemistry

The hydrogel constructs undergoing chondrogenic differentiation were harvested on day 21 and fixed in ROTI^®^Histofix 4% for 60 min, washed afterwards in PBS, and incubated in Tissue Tek^®^ O.C.T. (Sakura Finetek, Torrance, CA, USA) overnight at 4 °C. The next day, the constructs were transferred into fresh O.C.T., shock frozen in liquid nitrogen, and stored at −20 °C until the cryosection procedure. Longitudinal sections of 8 μm thickness were prepared and collected on Super Frost^®^ plus glass slides (R. Langenbrinck, Emmendingen, Germany).

For histology, the samples were stained with Weigert’s hematoxylin, fast green, and safranin O to analyze the distribution of GAG [47], and with Weigert’s hematoxylin and picrosirius red to stain for collagen distribution [48].

For immunohistochemistry, the sections were washed in ddH_2_O, and antigen retrieval was performed using proteinase K for 5 min at RT. The blocking was performed with 1% bovine serum albumin (BSA) in PBS for 30 min and primary antibodies were diluted in 1% BSA in PBS and incubated overnight in a humidified chamber at RT. Antibodies against collagen type II (II-4C11, 1:100, Sigma-Aldrich, St. Louis, MO, USA) and aggrecan (969D4D11, 1:300, Thermo Fisher Scientific, Waltham, MA, USA) were used. Sections were washed three times in PBS, and secondary antibodies were diluted in 1% BSA in PBS and applied in the dark for 1 h. A goat anti-mouse (Alexa Fluor 488, 1:100, (115-545-068, Jackson ImmunoResearch, Dianova, Hamburg, Germany)) secondary antibody was used. Finally, the slides were washed three times in PBS and mounted with DAPI mounting medium ImmunoSelect^®^. Images were taken with a fluorescence microscope (Axio Observer Z1, equipped with epifluorescence optics, an MRm camera, and an Apotome, Carl Zeiss, Jena, Germany).

### 4.15. Biochemical Analyses

For analyses of DNA, GAG, and collagen content the constructs were digested in 0.5 mL of a papain solution (3 U/mL) for 16 h at 60 °C. Prior to digestion, the constructs were homogenized at 25 Hz for 5 min using a TissueLyser LT (Qiagen, Hilden, Germany).

The DNA content of the hydrogels was determined employing a Quant-iT™PicoGreen^®^ dsDNA Reagent and Kit (Thermo Fisher Scientific, Waltham, MA, USA), according to the manufacturer’s instructions. The DNA quantification was carried out fluorometrically at λ_ex_ = 485 nm and λ_em_ = 538 nm, using a microplate reader (Spark 20M, Tecan, Maennedorf, Switzerland) and a Lambda DNA standard. The amount of produced GAG was measured using the DMMB assay. The GAG amount was determined with a spectrophotometer at 525 nm, using bovine chondroitin sulfate as standard [49]. The content of hydroxyproline was measured, after acid hydrolysis and reaction with DAB and chloramine T. The quantification was carried out with a spectrophotometer at 570 nm, using L-hydroxyproline as standard [50,51].

### 4.16. Statistics

Sigma Plot 12.5 (Systat Software GmbH, Erkrath, Germany) was used for statistical analysis and Origin 2018b (OriginLab Corp., Northampton, MA, USA) for graphical plots. Data are reported as the mean and standard deviation from at least three replicates, if not stated otherwise. For GAG and collagen production, multiple groups were compared using one-way ANOVA with Tukey’s post hoc test. Two-way ANOVA with Tukey’s post hoc test was used with time (days 1 and 7) and blend hydrogel contents (3%, 4%, and 5%) for cell viability. Significant differences are marked as follows: * *p* < 0.05, ** *p* < 0.01, and *** *p* < 0.001.

## 5. Conclusions

In this study, we developed a novel synthesis path for a new HA-based material for in situ formation of biocompatible hydrogels. We showed that the degree of oxidation by the TEMPO/TCC oxidation can be further increased by using anhydrous DMF as the solvent to avoid overoxidation of the primary alcohol group to carboxylic acid functions. With the higher DO, we were able to generate long-term and mechanical stable hydrogels without the need for secondary crosslinking, even in cell culture medium. By using the reversible Schiff base crosslinking, we could abstain from UV exposure in cell culture and achieved good cell viability. The formed hydrogels were successfully applied for the chondrogenic differentiation of hMSC, where the newly formed ECM was essential to retain the hydrogel structure during the long-term culture. Due to the very fast gelation and high mechanical stiffness, this still reversible hydrogel is not immediately suitable for extrusion 3D printing, where a gelation on a longer time scale would be desired. Nevertheless, casting of this fast crosslinked hydrogel in more complex prefabricated shapes could be applied to also encapsulate cells in different geometries. Additionally, this system may be used for the formation of microgels using microfluidic two component mixers with the encapsulation of cells.

## Figures and Tables

**Table 1:** DO in %, weight and number average molecular weights (M_w_ and M_n_ in kDa) of native HA, oxHA, and various batches of proxHA synthesized with 0.25 eq. of TCC. Grey marked batches were combined to one blend.

	DO (%)	M_w_ (kDa)	M_n_ (kDa)	Sample Name
Native HA	0	61	24	HA
proxHA	73.7	95	27	DO 73.7%
	63.2	36	11	DO 63.2%
	57.2	96	44	DO 57.2%
	55.0	75	33	DO 55.0%
	49.5	144	45	DO 49.5%
Blend	53.3	87	34	Blend
oxHA	11.7	260	121	oxHA

**Table 2:** Values of dissociation constant of unmodified HA, NaIO_4_ oxidized (oxHA), and TEMPO/TCC oxidized HA (proxHA) with different degrees of oxidation (DO).

Sample	M_w_ (kDa)	M_n_ (kDa)	DO (%)	K_D_ (nM)
HA	61	24	0	533 ± 87
oxHA	260	121	11.7	3546 ± 1052
proxHA	36	11	63.2	3330 ± 911

## Data Availability

Data is contained within the article or Supplementary Material.

## Supplementary Materials

The following are available online, Figure S1: Cell viability of in vitro cultured hMSCs in 3 wt% hydrogels (DO 73.7%) after day 1, 7, and 21, shown by live/dead staining. Viable cells were labeled green with calcein-AM and dead cells with EthD-I., Figure S2: Proportion of the hydrogels (3, 4, and 5 wt%) with proliferated cells over 3 weeks as compared with hydrogels with chondrogenic differentiated cells (3, 4, and 5 wt%), Table S1: Volumes and masses used for hydrogels for mechanical tests and investigation of degradation/swelling behavior, Table S2: Volumes and masses used for hydrogels for cell culture experiments.

## Sample Availability

Not available.
