# Supplementary material for: TEMPO/TCC as a Chemo Selective Alternative for the Oxidation of Hyaluronic Acid
Source: Molecules. 2021 Oct 1;26(19):5963. doi: 10.3390/molecules26195963 (PMC8512827; doi:10.3390/molecules26195963)
Supplement: Supplementary file 1 [file molecules-26-05963-s001.zip › molecules-1379593-supplementary.pdf]

Supplementary materials:

Cell Culture

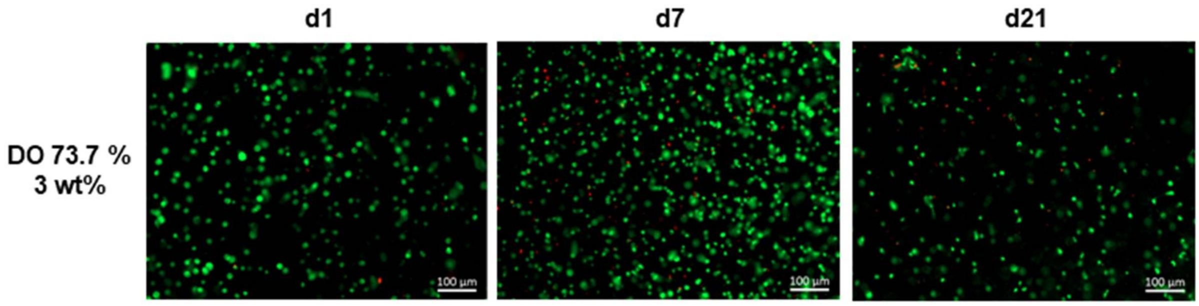

Figure S1: Cell viability of in vitro cultured hMSCs in 3 wt% hydrogels (DO 73.7 %) after day 1, 7 and 21, shown by live/dead staining. Viable cells were labeled green with calcein-AM and dead cells with EthD-I.

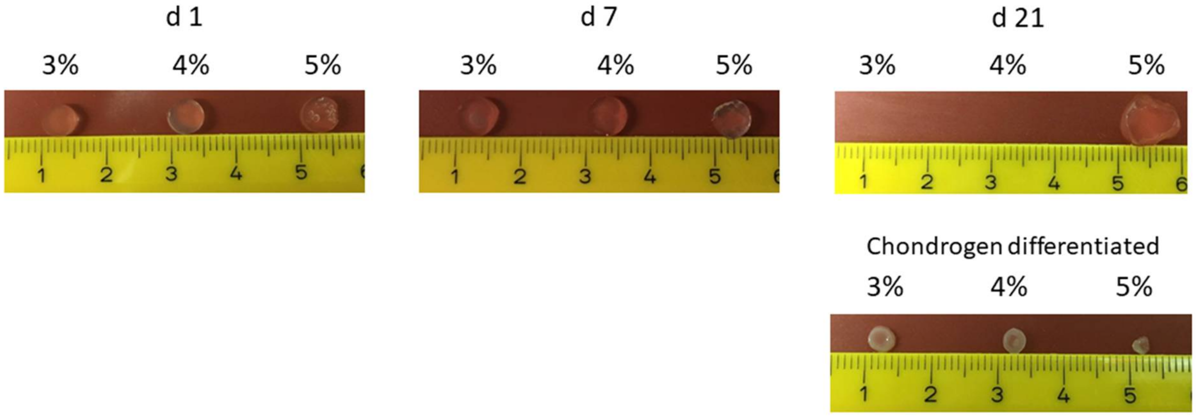

Figure S2: Dimensions of the hydrogels (3, 4, and 5 wt%) with proliferated cells over 3 weeks in comparison of hydrogels with chondrogenic differentiated cells (3, 4 and 5 wt%).

Preparation of Hydrogels

All hydrogels were prepared as described in chapter 2.9 with following voluminal and masses.

Table S1: Volumina and masses used for hydrogels for mechanical tests and investigation of degradation/swelling behavior.

wt%	DO [%]	m(proxHA) [mg]	V(PBS)	V(ADH 2 wt%)
	49.5		385.3	74.7
	55.0		377.0	83.0
3	57.2	14.2	373.7	86.3
	73.7		348.8	111.2
	53.5 (Blend)		379.6	80.4
2	53.5 (Blend)	9.3	353.0	107.0 (ADH 1 wt%)
4	53.5 (Blend)	19.2	350.5	109.5
5	53.5 (Blend)	24.2	321.4	138.6

Table S2: Volumina and masses used for hydrogels for cell culture experiments.

wt%	DO [%]	m(proxHA) [mg]	V(PBS)	V(ADH 2 wt%)

3	73.7	23.2	568.7	181.3
3	53.5 (Blend)	23.2	617.8	132.2
4	53.5 (Blend)	31.3	572.0	178.0
5	53.5 (Blend)	39.5	525.1	225
